# Effects of Presentation Type and Visual Control in Numerosity Discrimination: Implications for Number Processing?

**DOI:** 10.3389/fpsyg.2016.00066

**Published:** 2016-02-01

**Authors:** Karolien Smets, Pieter Moors, Bert Reynvoet

**Affiliations:** ^1^Brain and Cognition, Faculty of Psychology and Educational Sciences, KU LeuvenLeuven, Belgium; ^2^Faculty of Psychology and Educational Sciences, KU LeuvenKortrijk, Belgium

**Keywords:** ANS, methodology, comparison, presentation format, visual cue control, simultaneous, sequential

## Abstract

Performance in a non-symbolic comparison task in which participants are asked to indicate the larger numerosity of two dot arrays, is assumed to be supported by the Approximate Number System (ANS). This system allows participants to judge numerosity independently from other visual cues. Supporting this idea, previous studies indicated that numerosity can be processed when visual cues are controlled for. Consequently, distinct types of visual cue control are assumed to be interchangeable. However, a previous study showed that the type of visual cue control affected performance using a simultaneous presentation of the stimuli in numerosity comparison. In the current study, we explored whether the influence of the type of visual cue control on performance disappeared when sequentially presenting each stimulus in numerosity comparison. While the influence of the applied type of visual cue control was significantly more evident in the simultaneous condition, sequentially presenting the stimuli did not completely exclude the influence of distinct types of visual cue control. Altogether, these results indicate that the implicit assumption that it is possible to compare performances across studies with a differential visual cue control is unwarranted and that the influence of the type of visual cue control partly depends on the presentation format of the stimuli.

## Introduction

It is commonly assumed that an innate system exists that enables humans and non-human species to compare non-symbolic numerosities (e.g., arrays of dots): the Approximate Number System (ANS; Dehaene, 1997; Feigenson et al., 2004). The acuity of the ANS varies from individual to individual (Halberda et al., 2008) and can be measured by means of a comparison task. In such a task, participants are instructed to indicate the larger of two presented numerosities (Buckley and Gillman, 1974; Piazza et al., 2010), leading to ratio-dependent performance. An estimate of ANS acuity can subsequently be obtained by calculating either the individual Weber fraction (Piazza et al., 2010) or the average accuracy (Gilmore et al., 2011), two measures that are strongly correlated (Lindskog et al., 2013b).

Computational accounts suggest that the ANS disposes over the robust capacity to extract pure numerosity independently from other co-varying visual cues, such as for instance the cumulative area or area extended by the dots. For instance, the neural model of Dehaene and Changeux (1993) implicates that visual cues are discounted or normalized, after which abstract numerosity can be extracted. In the deep network connectionist model of Stoianov and Zorzi (2012), two hierarchically organized layers of neurons emerged after unsupervised learning. Neurons in the first layer (i.e., center-On local detectors) were found to encode high spatial frequency information from the initial visual input. Numerosity detectors in the second layer of the model combined the high spatial frequency representation of the image with an inhibitory signal representing cumulative area. As a consequence, pure numerosity independent from visual cues can be extracted (Cappelletti et al., 2014).

A wide range of methodological variables can be differently manipulated within the design of the frequently used comparison task, resulting in several variants of this non-symbolic number processing task. Some researchers implicitly assume that these methodological differences do not affect obtained results. Therefore, results from studies with distinct methodological characteristics are combined to for instance demonstrate the developmental trajectory of ANS acuity (see Figure 4 in Halberda and Feigenson, 2008; Figure 3 in Piazza et al., 2010). The implicit assumption behind this reasoning, posing the possibility to compare results with differing methodologies, may however be unwarranted. As Inglis and Gilmore (2014) indicated, it is unreasonable to assume that Weber fractions and accuracies are independent from methodological characteristics. This implicit assumption of interchangeable methodologies is also reflected in researchers comparing their results with previous research employing fundamentally different methods. For instance, several researchers relate obtained results with respect to a relationship between non-symbolic number processing and mathematics to other studies that measured non-symbolic number processing in a different and potentially incomparable manner (e.g., Castronovo and Göbel, 2012; Halberda et al., 2012). Szücs et al. (2013) reached a similar conclusion with respect to the incomparability of different studies: They suggested that Weber fractions from studies with distinct methodological characteristics cannot be readily compared as these Weber fractions may not reflect pure numerosity processes, but are alternatively confounded by other variables.

Recently, there is an increased awareness with regard to the effects of methodological differences. For instance, entirely different tasks, all assumed to measure the ANS, have been shown to lead to incomparable results (i.e., comparison and same-different in Smets et al., 2013; comparison, same-different and change detection in Smets et al., 2013). Even within the framework of a single task, methodological aspects that are differently manipulated can influence the results significantly. For instance, Inglis and Gilmore (2013, 2014) indicated that stimulus duration and the manipulated ratios can affect performance, making comparisons across studies that differ with respect to these variables difficult. However, one important methodological difference between studies using numerosity comparison has not been addressed in great detail before: the different precautions that are taken to ensure that participants are unable to base their responses on the visual cues that accompany numerosities. Numerosities and visual cues correlate strongly in the majority of instances in everyday life, usually because similar objects need to be compared. However, for researchers to be able to study pure numerosity processing, human participants (e.g., Pica et al., 2004) but also animals (e.g., Pisa and Agrillo, 2009; Agrillo et al., 2011) need to be discouraged to base their responses on cues other than numerosity. To achieve this, several different methods that aim at controlling visual cues have been developed (e.g., Rousselle et al., 2004; Dehaene et al., 2005; Ansari et al., 2007; Gebuis and Reynvoet, 2011a; Price et al., 2012). Considering it is virtually impossible to discuss and examine all of these distinct methods to control visual cues of dot arrays, our focus will be on two of them to address the potential issue. By means of the first of these two methods, dot arrays are created in which in half of the trials the visual cues “diameter of the dots” and “area extended by the dots” (or convex hull) are designed to be maintained at a constant level, while consequently the visual cue “surface” (the sum of the individual dot surfaces) co-varies with numerosity. The more numerous dot array is thus supposed to be characterized by a larger surface than the smaller numerosity to be able to keep dot diameter and area extended constant between both numerosities that need to be compared (e.g., Dehaene et al., 2005). Hence, in these trials, surface is congruent or co-varying with numerosity. In the other half of the trials, this is vice versa: To keep surface at a constant level, dot diameter and area extended are allowed to co-vary with numerosity: The more numerous dot array of the number pair will be characterized by a smaller average dot size and a larger area extended than the smaller numerosity to be able to maintain surface between both dot arrays at a constant level. Hence, in these trials, dot size and area extended are congruent or co-varying with numerosity. This method is assumed to be an appropriate control for visual cues if and only if participants rely on a *single* visual cue when judging number and not for instance switch between multiple visual cues (Gebuis and Reynvoet, 2012; Szücs et al., 2013). Considering this method only takes into account a few visual cues, we will refer to this method and related methods as simple sensory control methods. The simple sensory control method has been used in a number of previous studies investigating non-symbolic number processing (e.g., Sasanguie et al., 2011; Piazza et al., 2013).

Gebuis and Reynvoet (2011a) suggested that simple sensory control methods may not be sufficient if participants for instance switch between several visual cues or integrate information from multiple visual cues. These authors therefore established an alternative method. When constructing dot arrays according to their method (Gebuis and Reynvoet, 2011a), multiple visual cues are taken into account to ensure that these are uninformative about numerosity across trials: (a) “area extended by the dots” (convex hull or the smallest contour that can be drawn around the dots), (b) “surface” (aggregate value of the different dot surfaces within one array), (c) “diameter of the dots,” (d) “circumference,” and (e) “density” (surface divided by the area extended by the dots). Thus, this method is assumed to be an appropriate control when participants switch between or integrate multiple visual cues, which is why we refer to this method as a multi-sensory control method. By means of this multi-sensory control method, area extended by the dots and dot diameter are manipulated in such a way that both are larger in half of the trials and smaller in the other half of the trials for the more numerous array, while dot sizes are drawn from a less skewed distribution for the congruent trials (i.e., trials in which visual cues provide reliable information with respect to numerosity; e.g., a more numerous array that is characterized by a larger convex hull) as opposed to the incongruent trials (i.e., trials in which visual cues provide contradictory information with respect to numerosity; e.g., a larger numerosity characterized by a smaller convex hull) (see Gebuis and Reynvoet, 2011a for a more detailed description of this method). As a result of these manipulations, the difference between two stimuli in either of the visual cues (area extended, surface, diameter, density and circumference) does not correlate with the difference between their respective numerosities across trials. Furthermore, this can be explicitly verified by performing regression analyses on the specific values for each of the visual cues, provided when utilizing the method of Gebuis and Reynvoet (2011a).

These and other methods are generally treated as interchangeable methods to control visual cues and/or assumed to be irrelevant by the majority of researchers in the field as long as there is *some* type of control of these interfering visual cues. In a recent study (Smets et al., 2015) however, we decided to specifically contrast the two aforementioned methods to control visual cues of dot arrays (i.e., the simple vs. the multi-sensory control method; Dehaene et al., 2005; Gebuis and Reynvoet, 2011a) in two distinct tasks: numerosity comparison and estimation. For the comparison task, the results indicated significantly different and unrelated accuracies and Weber fractions with a better performance when visual cues were controlled according to the simple sensory control method (Dehaene et al., 2005). In correspondence with the suggestion made by Szücs et al. (2013) who found a substantially larger Weber fraction when using multi-sensory control compared to previous research, we reasoned that the difference between the two types of visual cue control was due to the more stringent nature of controlling visual cues with the multi-sensory control method. More concrete, visual cues of stimuli in the multi-sensory control condition are less informative and more ambiguous with respect to numerosity, ultimately leading to a decreased performance in this condition. The results with respect to the comparison task were in sharp contrast with the results obtained by means of the numerosity estimation task (Smets et al., 2015). In this task, participants were presented with a dot array and subsequently instructed to estimate how many dots were present by providing a symbolic label. The distinct visual cue controls did not influence the performance in this task: Not only was estimation performance rather similar in the two visual cue control conditions, performances were also significantly related. A potential reason for an apparent lack of an effect of the type of visual cue control that was used in numerosity estimation may be related to the fact that participants were specifically required to provide a number symbol in response to the dot array. This particularly focuses attention on numerosity, potentially diminishing the influence of the type of visual cue control that is applied. However, considering that Gebuis and Reynvoet (2012) showed that visual cues still influence performance on a more stimulus-related level, the effect of visual cues in general is most probably not entirely absent in the estimation task. Nevertheless, we concluded that in some instances more than in others, the influence of different types of visual cue control is *more* evident: The simultaneous comparison task in which both stimuli are presented at the same time on different sides of the screen, used in both Szücs et al. (2013) and Smets et al. (2015), may prompt participants (more) to experience a potentially disrupting influence from visual cues or to (un)consciously rely on these cues (see also Rousselle et al., 2004), compared to for instance an estimation task. This will eventually lead to differences between distinct types of visual cue control that differ in the rigor of this control.

Comparison tasks with simultaneous presentation of the stimuli are frequently used in the literature (e.g., Gilmore et al., 2013; Inglis and Gilmore, 2013; Smets et al., 2013). However, other potential presentation formats are also possible. For instance, instead of presenting the stimuli simultaneously side by side, they can also be presented sequentially in the same location (e.g., Ansari et al., 2007; Hayashi et al., 2013) or simultaneously intermixed with different colors (e.g., Ansari et al., 2007; Halberda et al., 2012; Lindskog et al., 2013a). Previous research indicated that comparison performance in general can be affected by a change in presentation format. For instance, Price et al. (2012) found that performance was significantly higher with a sequential presentation of the stimuli compared to an intermixed presentation format. The difference between the sequential and the simultaneous presentation format in which both stimuli are presented in parallel however did not reach significance in that particular study (Price et al., 2012).

Despite the finding of Price et al. (2012), psychophysical research outside the numerical cognition domain (Brown and Rebbin, 1970; Frick, 1985) suggests that a simultaneous paired vs. sequential presentation format could in fact be an important factor that may lead to incomparable effects/performances and a lack of validity, as is the case with entirely different tasks (Smets et al., 2014). Specifically and of importance for the topic of the current study, these studies suggest that the influence of visual cues and thus of the applied type of visual cue control may manifest itself differently when simultaneously vs. sequentially presenting stimuli. More concrete, simultaneously presenting stimuli in numerosity comparison allows explicit and refined comparisons of visual stimuli (Brown and Rebbin, 1970) and necessitates visuo-spatial short term memory (Frick, 1985). In addition, simultaneous comparison permits participants to attend to the stimuli in the most straightforward manner possible (Crowder, 1966). The beneficent and rather simple comparison of visual aspects in simultaneous comparison may be responsible for the observed influence of visual cues: Previous research indicated that visual cues are extracted automatically (Clearfield and Mix, 2001; Hurewitz et al., 2006; Gebuis and Reynvoet, 2013), and are thus likely to influence performance in easy visual simultaneous comparison. Consequently, distinct types of visual cue control that differ in the level of information they provide with respect to numerosity, will influence performance. Whereas processing visual characteristics of the stimuli is evident in simultaneous comparison, sequential presentation of stimuli however, does not so readily lend itself to detailed visual comparison (Brown and Rebbin, 1970). Furthermore, visuo-spatial short term memory is not required with this presentation format (Frick, 1985). More specifically, only the last display in the sequence can be kept in visuo-spatial short term memory. The fact that the first stimulus in the sequence cannot be retained in visuo-spatial short term memory as efficiently necessitates the use of another strategy. A potential strategy could be to extract numerosity on each sequential stimulus and compare these, especially because “number” is emphasized in the task instructions. The use of such a strategy appears rather similar to what occurs in numerosity estimation, for which our results pointed toward a diminished influence of distinct visual cue controls (Smets et al., 2015). Hence, because a direct comparison of visual cues is less evident in sequential comparison, participants are expected to be less biased by visual cues and thus by distinct visual cue controls.

This possibility was explicitly explored in the current study by orthogonally manipulating both the type of visual cue control (according to the two methods described above: simple vs. multi-sensory control) and the presentation format (simultaneously paired vs. sequential) within the same participants. By contrasting the two types of visual cue control, the following question is addressed: Is the influence of the specific type of visual cue control in numerosity comparison excluded or less evident when the stimuli are presented in a sequential instead of a simultaneous fashion? More general, this study also relates to the question whether certain methodological characteristics of non-symbolic number tasks kindle a stronger influence of the applied type of visual cue control than others, as suggested by the results of Smets et al. (2015).

## Materials and methods

### Participants

Forty adults participated in the present study (mean age = 21 years, *SD* = 2.77 years, 31 females) and performed all four conditions which were administered in separate blocks. They either received course credit or were paid for their participation. The Ethical Committee of the Faculty of Psychology and Educational Sciences of the University of Leuven approved the experiment. All participants gave written informed consent.

### Stimuli and procedure

**Figure 1:**
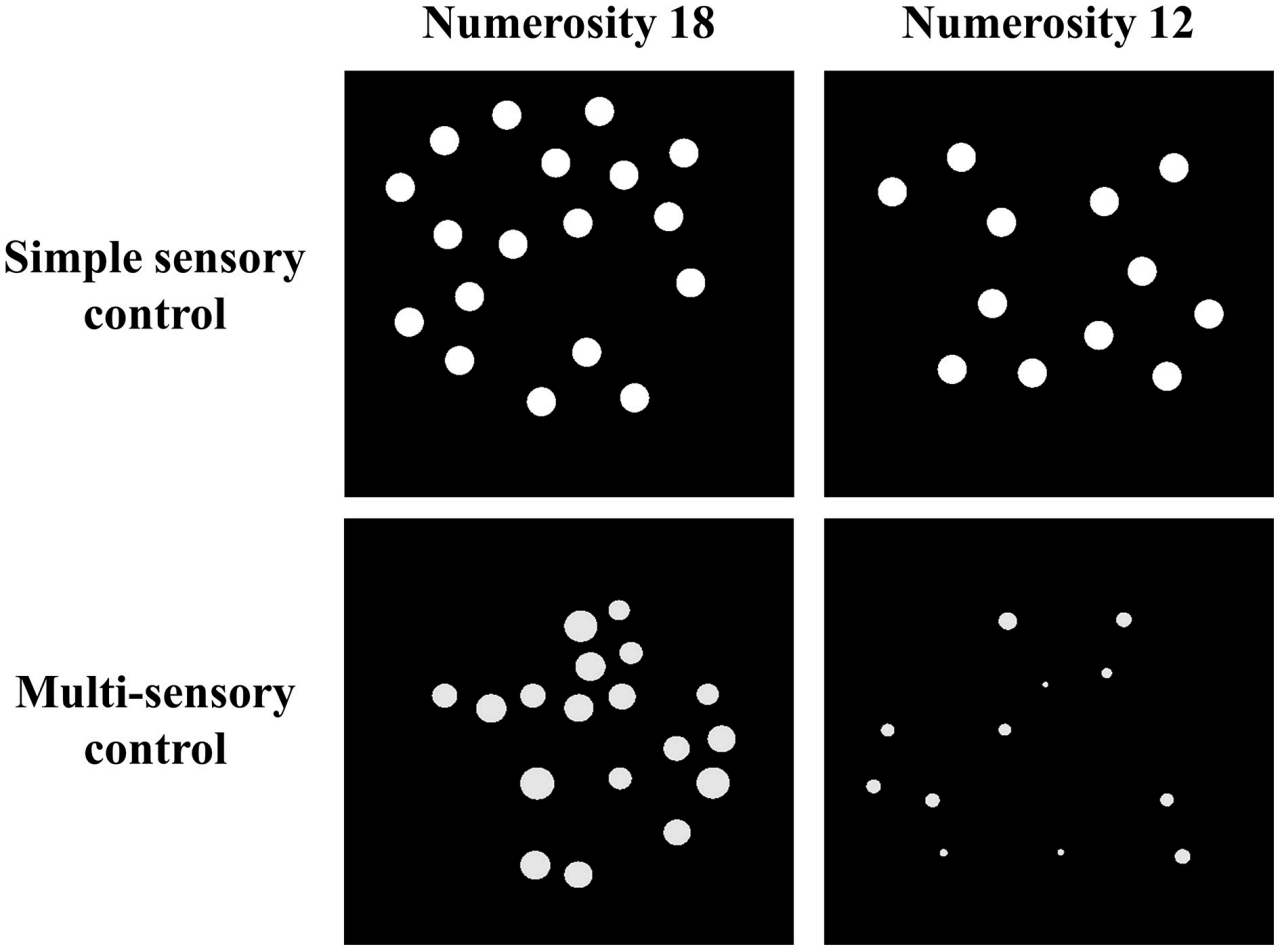
**Examples of one stimuli pair (i.e., 18–12) for the simple sensory control condition (example of 50% of the trials in which dot diameter is kept constant, while in the other 50% of the trials surface is constant, constructed with Dehaene et al., 2005) and the multi-sensory control condition (constructed with Gebuis and Reynvoet, 2011a)**.

Stimuli were white dots on a black background and were presented on a 17-inch color screen by means of E-prime 1.1 (Psychology Software Tools, http://www.pstnet.com). The dot arrays (on average 8.89° visual angle) were presented either sequentially in the same location or simultaneously in parallel on both sides of the screen. The method that was used to construct these dot arrays was manipulated as a within-subjects variable: Dot arrays were either created according to a simple sensory control method (script of Dehaene et al., 2005; http://dx.doi.org/10.6084/m9.figshare.1418022) or a multi-sensory control method (script of Gebuis and Reynvoet, 2011a; http://dx.doi.org/10.6084/m9.figshare.1418023). By means of the first method, dot arrays are constructed in which (a) dot diameter and area extended by the dots is maintained at a constant level while surface is congruent with numerosity, or (b) dot diameter and area extended by the dots are congruent with numerosity and surface is kept constant between both numerosities that need to be compared. This method is assumed to be an appropriate control when participants rely on a single visual cue to compare numerosities (Gebuis and Reynvoet, 2012) and as it manipulates only a few visual cues, we refer to this condition as the “simple sensory control condition.” By means of the second method (i.e., the multi-sensory control method of Gebuis and Reynvoet, 2011a), multiple visual cues are manipulated and controlled, making them uninformative about numerosity across trials: (a) “area extended by the dots,” (b) “surface,” (c) “dot size,” (d) “circumference,” and (e) “density.” We refer to the condition in which dot arrays are created according to this method as the “multi-sensory control condition” because it is constructed as an appropriate control for visual cues when participants switch between several cues or integrate multiple visual cues in a single trial. A stimuli example of the respective visual cue control conditions is shown in Figure 1 (see http://dx.doi.org/10.6084/m9.figshare.1425407 and http://dx.doi.org/10.6084/m9.figshare.1425405, respectively for all (numerical and visual) parameters of the stimuli in the multi-sensory control condition and the single sensory control condition). In addition to manipulating the type of visual cue control, presentation format of the stimuli was also manipulated: Stimuli were presented either simultaneously on both sides of the screen (with an average visual angle of 10.16° between both stimuli) or sequentially in the same location. The orthogonal manipulation of presentation format and type of visual cue control prompted four conditions within-participants: (a) a simultaneous simple sensory control condition, (b) a simultaneous multi-sensory control condition, (c) a sequential simple sensory control condition, and (d) a sequential multi-sensory control condition. These conditions were administered in separate blocks.

In all conditions, participants were instructed to indicate the larger of two presented numerosities. One numerosity was always “18,” while the other numerosity was smaller than this referent in half of the trials (12, 13, and 15) and larger in the other half of the trials (22, 25, and 27), leading to three different ratios between the numerosities (1.2, 1.4, and 1.5). We opted to include these and not more difficult ratios, because we expected performance in the multi-sensory control condition to suffer from the stringent control of visual cues as suggested by Szücs et al. (2013) and in correspondence with the results of Smets et al. (2015).

**Figure 2:**
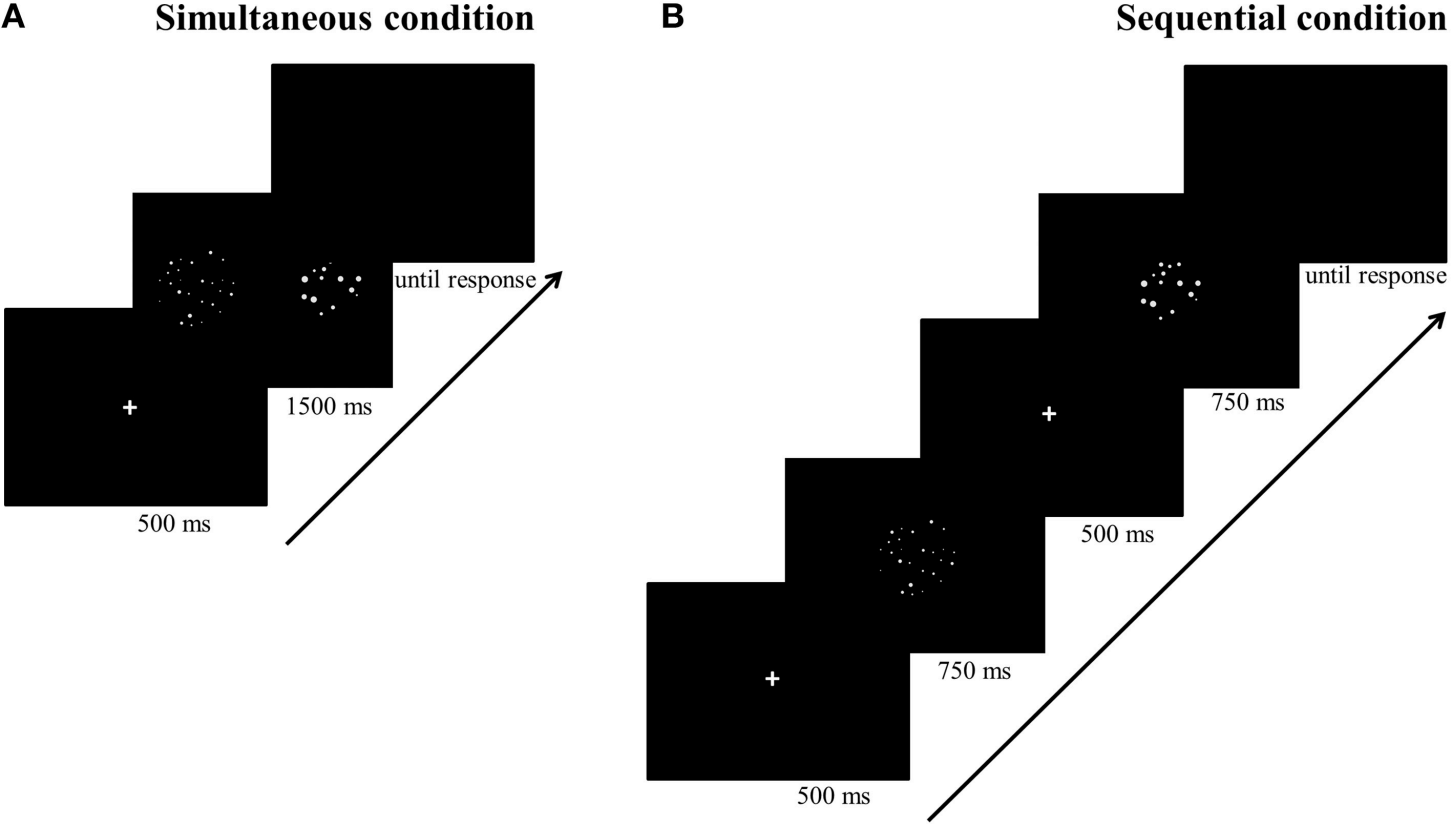
**Procedure of a trial in the simultaneous (A) and sequential (B) conditions**.

For the simultaneous conditions (see Figure 2A), each trial started with a fixation cross for 500 ms, followed by the two dot arrays which were simultaneously presented, one on each side of the screen, for 1500 ms. Afterwards, a blank was displayed until the participant responded. Participants could either respond during the presentation of the dot arrays or after they disappeared. For the sequential conditions (see Figure 2B), a fixation cross was also presented for 500 ms at the start of each trial. Afterwards, the first dot array was presented for 750 ms, after which a fixation cross was presented for 500 ms to call participant's attention that a new stimulus would be presented. Next, the second dot array was presented for 750 ms at the same location, followed by a blank until a response was registered. Participants could either answer during the presentation of the second dot array or when the blank was displayed. The presentation times of the sequentially presented stimuli were each half of the presentation time of the stimuli presented simultaneously to match total presentation time of stimuli in both conditions. We took this precaution to permit participants the same amount of time to process the numerosities in both conditions (as opposed to for instance Price et al., 2012, in which a certain advantage for the sequential condition may have been present as a consequence of chosen presentation times). Although the total presentation time might seem long (but see for instance Price et al., 2012; Szücs et al., 2013) counting all dots is nearly impossible within this time span because the stimuli ranged from 12 to 27 dots. Moreover, participants were explicitly instructed to respond as quickly and as accurately as possible without counting the dots.

In all conditions, each number pair was repeated 16 times. This resulted in 96 trials per ratio condition (3 ratios ^*^ 2 lower/higher than the referent ^*^ 16 repetitions) and 384 trials in total (4 conditions ^*^ 96 trials per task condition). Participants were administered 10 practice trials in each of the four conditions. The order in which participants performed the four conditions was counterbalanced, although the two sequential conditions and the two simultaneous conditions were always conducted one after another (i.e., in pairs) to avoid too much confusion on the participants' part. Within each condition, participants received trials in a random order and each task condition lasted approximately 8 min, leading to a total testing time of 32 min (= 4^*^ 8).

### Data analyses

In a first analysis, we assessed reliability in accuracy by computing the split-half reliability for each of the four conditions, using average accuracies per condition across ratios. Next, we calculated both participants' mean accuracies and median reaction times (for correct trials only) per ratio and per condition and submitted these to a repeated measures ANOVA with ratio (three levels: 1.2, 1.4, and 1.5), presentation format (two levels: sequential and simultaneous) and type of visual cue control (two levels: simple sensory and multi-sensory control) as within-subjects factors, for accuracies and reaction times separately. In addition to these variables, we computed participants' individual Weber fractions (*w*) in each of the four conditions by fitting the data by means of the following function (Halberda et al., 2008; Piazza et al., 2010):

$$\begin{array}{l} \text{Proportion}\;\text{Judged}\;\text{Larger}\;\left(n1,n2\right)\;\\ \;=\frac{1}{2}\cdot \text{erfc}\left(\frac{n2-n1}{\sqrt{2}w\sqrt{n1^{2}+n2^{2}}}\right) \end{array}$$

where *n*1 refers to the numerosity that needs to be discerned from the reference numerosity *n*2, and erfc is the complementary error function. Decision curves were defined for each individual participants for all values of *w* between 0.01 and 10 in steps of 0.01. Next, a least squares algorithm determined the curve which fitted the data of each individual participant the best (see also Szücs et al., 2013 for a similar procedure), making it possible to infer the corresponding Weber fraction in each condition. Two participants with extreme Weber fractions in one of the conditions (>3 *SD*) were excluded from further analyses. The individual Weber fractions were submitted to a repeated measures ANOVA with presentation format (two levels: simultaneous vs. sequential) and visual cue control (two levels: simple sensory and multi-sensory control) as within-subjects variables. When the assumption of sphericity was violated in any of the repeated measures analyses, *p*-values were corrected by means of the Greenhouse-Geisser method (pGG). Finally, correlations between conditions were calculated for accuracies and Weber fractions. Similar correlations between reaction times were not computed as these could not be controlled for differences in general processing speed.

## Results

### Reliabilities

Split-half reliability in accuracy for each of the four conditions was calculated. These analyses indicated low to moderate reliabilities between 0.48 and 0.73. However, reliabilities are in agreement with what has been obtained in previous research with a similar number of trials (e.g., Maloney et al., 2010; Price et al., 2012). In addition, split-half reliabilities for Weber fractions were also calculated and ranged between 0.37 and 0.68.

### Accuracies

**Figure 3:**
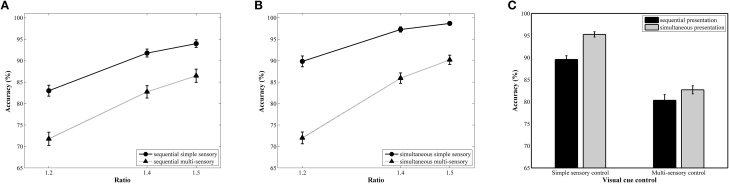
**Accuracy results for each ratio in the simultaneous (A) and sequential (B) conditions with simple and multi-sensory control**. The signature ratio effect was present in all conditions. In **(C)**, the effect of a distinct visual cue control on average accuracy is illustrated for both presentation conditions. Differences in average accuracy between the simple and multi-sensory control were significant in both the simultaneous and sequential condition, but the difference in performance was significantly smaller in the latter.

The repeated measures ANOVA with accuracy as the dependent variable revealed a significant main effect of ratio, *F*_(2, 78)_ = 257.81, *pGG* < 0.001, $\eta_{p}^{2}=0$.87. A follow-up linear contrast indicated a significant increase in accuracy with increasing ratio, *F*_(1, 39)_ = 497.06, *p* < 0.001, $\eta_{p}^{2}=0$.93 (81, 89, and 92%, respectively; see Figure 3A for the ratio effect in the simultaneous conditions and Figure 3B for the ratio effect in the sequential conditions). The main effects of presentation format, *F*_(1, 39)_ = 25.78, *p* < 0.001, $\eta_{p}^{2}=0$.40, and type of visual cue control, *F*_(1, 39)_ = 190.98, *p* = < 0.001, $\eta_{p}^{2}=0$.83, were also significant. These factors were included in a significant interaction, *F*_(1, 39)_ = 5.69, *p* = 0.02, $\eta_{p}^{2}=$ 0.13. Pairwise *t*-tests within each presentation format indicated significant differences between the two types of visual cue control in both the simultaneous, *t*_(39)_ = 12.97, *p* < 0.001, *d* = 3.34, and the sequential condition, *t*_(39)_ = 8.21, *p* < 0.001, *d* = 1.74, with a better performance in the simple sensory control condition (*M* = 95%, *SD* = 3.76%, and *M* = 90%, *SD* = 5.31%, respectively) compared to the multi-sensory control condition (*M* = 83%, *SD* = 5.86%, and *M* = 80%, *SD* = 8.05%, respectively; see Figure 3C). Although an influence of the type of visual cue control is present in both presentation conditions, the effect sizes (Cohen's *d*) suggest a stronger impact of type of visual cue control when simultaneously presenting stimuli. This was further validated by calculating difference scores: We subtracted performance on the multi-sensory control condition from performance on the simple sensory control condition for both the simultaneous and sequential conditions. Hence, the difference score can be viewed as an indicator of the size of the effect we are interested in (i.e., the difference in performance between both visual cue control types). A subsequent *t*-test between the calculated difference scores of the simultaneous and sequential condition illustrated that this difference score is significantly different in the two presentation conditions, *t*_(39)_ = 2.39, *p* = 0.02, *d* = 0.76 (simultaneous: *M* = 12.56%, *SD* = 6.12%; sequential: *M* = 9.25%, *SD* = 7.12%), further validating our claim of a larger difference between the simple and multi-sensory control condition in the simultaneous compared to the sequential condition. The repeated measures analysis further indicated that the interaction between visual cue control and ratio was also significant, *F*_(2, 78)_ = 15.63, *pGG* < 0.001, $\eta_{p}^{2}=$ 0.29. Separate linear contrasts for each visual cue control condition indicated a significant increase in accuracy with increasing ratio in both control conditions, all *F*s > 147.63 and all *p*s < 0.001, all $\eta_{p}^{2}$s > 0.79 (simple sensory control condition: 86, 95, and 96%; multi-sensory control condition: 72, 84, and 88%), suggesting the presence of a ratio effect in both the simple and multi-sensory control condition. Hence, the significant interaction is not caused by the absence of a ratio effect in either of the conditions. Instead, the interaction is most probably due to a difference in the strength of the ratio effect: The ratio effect appears much steeper in the multi-sensory control conditions compared to the simple sensory control conditions (Figure 3) The two-way interaction between presentation and ratio and the three-way interaction did not reach significance, all *F*s < 2.76 and all *p*s > 0.07.

### Reaction times

**Figure 4:**
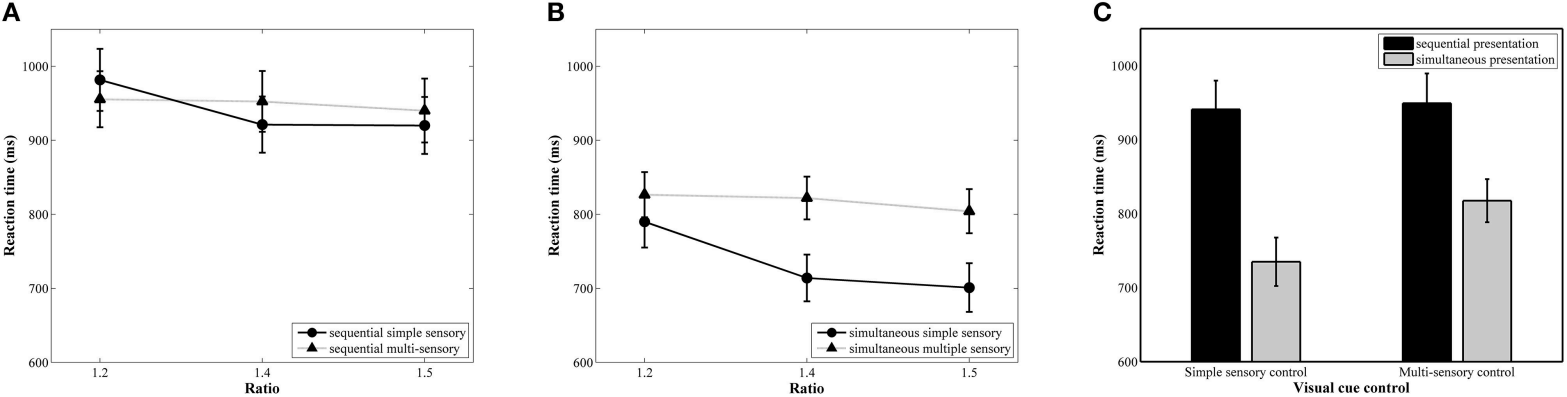
**Reaction time results for each ratio in the simultaneous (A) and sequential (B) conditions with simple and multi-sensory control**. The signature ratio effect was present in all conditions. In **(C)**, the effect of a distinct visual cue control on reaction time is illustrated for both presentation conditions. The difference in median reaction time between the simple and multi-sensory control was significant in the simultaneous, but not in the sequential condition.

The results of the repeated measures ANOVA with median reaction as dependent variable indicated a significant main effect of ratio, *F*_(2, 78)_ = 30.90, *p* < 0.001, $\eta_{p}^{2}=$ 0.44. A linear contrast indicated a significant linear decrease in reaction time with increasing ratio, *F*_(1, 39)_ = 52.77, *p* < 0.001, $\eta_{p}^{2}=$ 0.58 (888, 852, and 841 ms, respectively, see Figure 4A for the ratio effect in the simultaneous conditions and Figure 4B for the ratio effect in the sequential conditions). The main effects of presentation format, *F*(_1, 39)_ = 30.01, *p* < 0.001, $\eta_{p}^{2}=$ 0.44, and visual cue control, *F*_(1, 39)_ = 5.23, *p* = 0.03, $\eta_{p}^{2}=$ 0.12, were also significant. These factors were included in a marginally significant interaction, *F*_(1, 39)_ = 3.83, *p* = 0.055, $\eta_{p}^{2}=$ 0.09. Pairwise *t*-tests between the two visual cue control conditions within each presentation condition indicated that participants were faster to respond in the simple sensory control condition than in the multi-sensory control condition when the stimuli were presented simultaneously, *t*_(39)_ = 4.84, *p* < 0.001, *d* = 0.40 (simple sensory: *M* = 735 ms, *SD* = 207 ms; multi-sensory: *M* = 818 ms, *SD* = 184 ms), but reaction times did not significantly differ between these two control conditions when the stimuli were presented sequentially, *t*_(39)_ = 0.24, *p* = 0.81, *d* = 0.034 (simple sensory: *M* = 941 ms, *SD* = 246 ms; multi-sensory: *M* = 949 ms, *SD* = 253 ms; see Figure 4C). Difference scores were calculated by subtracting reaction time on the multi-sensory control condition from reaction time on the simple sensory control condition for both presentation formats. The *t*-test between the calculated difference scores of the simultaneous and sequential condition was marginally significant, *t*_(39)_ = 1.96, *p* = 0.057, *d* = 0.69 (simultaneous: *M* = −82.55 ms, *SD* = 108 ms; sequential: *M* = −8 ms, *SD* = 221 ms). The interaction between visual cue control and ratio also reached significance, *F*_(2, 78)_ = 17.58, *p* < 0.001, $\eta_{p}^{2}=$ 0.31. Follow-up separate linear contrasts for each control condition indicated a significant linear decrease in reaction time with increasing ratio in both control conditions, all *F*s > 4.42, all *p*s < 0.05 and all $\eta_{p}^{2}$s > 0.10. Figure 4 clarifies that the interaction is due to a difference in the size of the ratio effects between the two visual cue control conditions. The two-way interaction between presentation format and ratio and the three-way interaction did not reach significance, all *F*s < 1.35 and all *p*s > 0.27.

### Weber fractions

**Figure 5:**
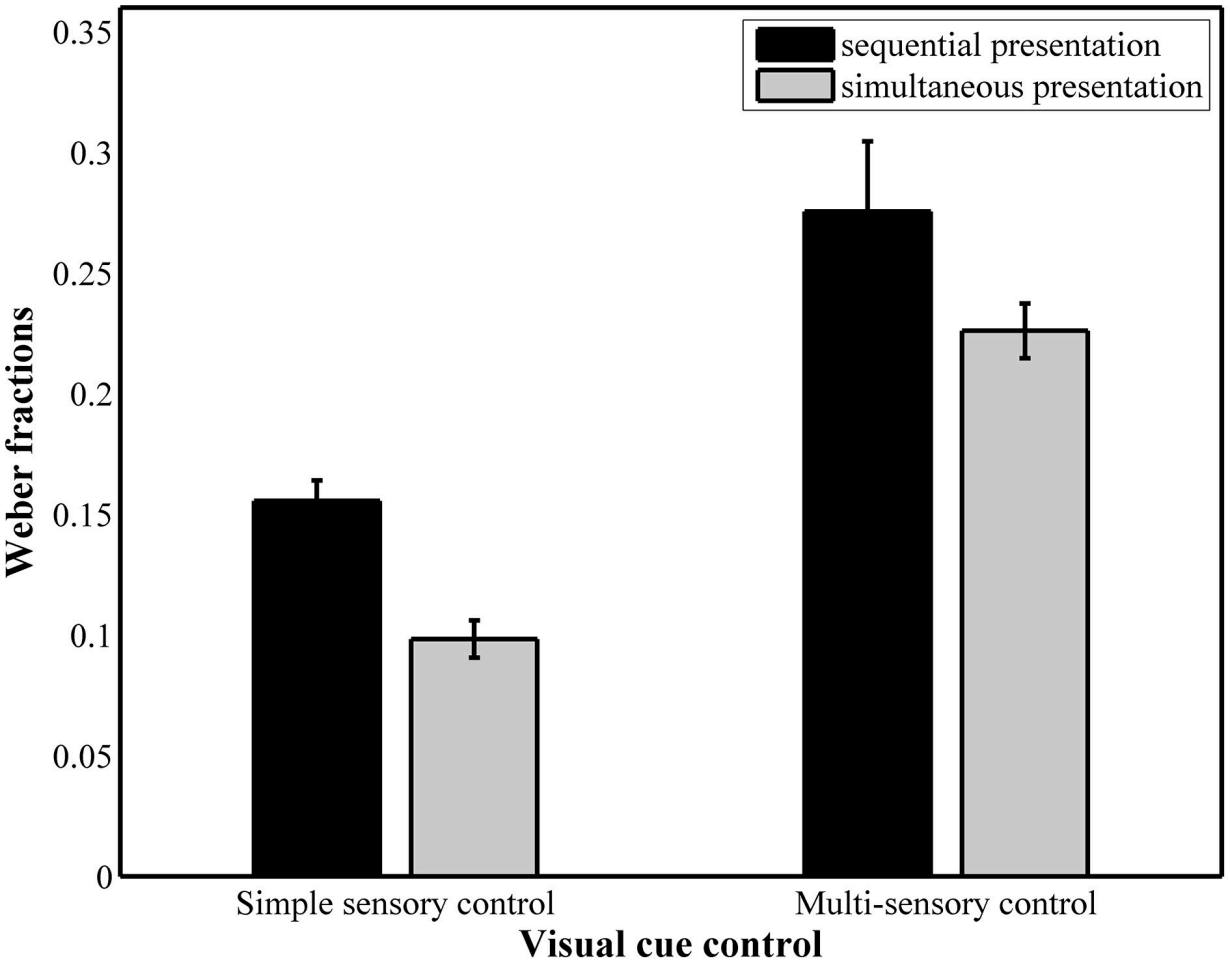
**Weber fractions for each of the four conditions**. The main effects of presentation format and visual cue control were significant.

The results of the repeated measures ANOVA with Weber fractions (Figure 5) as dependent variable pointed to a significant main effect of presentation format, *F*_(1, 37)_ = 17.71, *p*GG < 0.001, $\eta_{p}^{2}=$ 0.32, with a significantly higher Weber fraction in the sequential conditions (simple sensory: *M* = 0.15, *SD* = 0.047; multi-sensory: *M* = 0.25, *SD* = 0.096) compared to the simultaneous conditions (simple sensory: *M* = 0.10, *SD* = 0.048, multi-sensory: *M* = 0.22, *SD* = 0.059). The main effect of visual cue control was also significant, *F*_(1, 37)_ = 111.82, *p*GG < 0.001, $\eta_{p}^{2}=$ 0.75. Weber fractions were significantly higher in the multi-sensory control conditions (0.25 for sequential presentation and 0.22 for simultaneous presentation) than in the simple sensory control conditions (0.15 for sequential presentation and 0.10 for simultaneous presentation). The interaction between both presentation format and visual cue control did not reach significance, *F*_(1, 37)_ = 2.11, *p* = 0.16.

### Correlations

**Figure 6:**
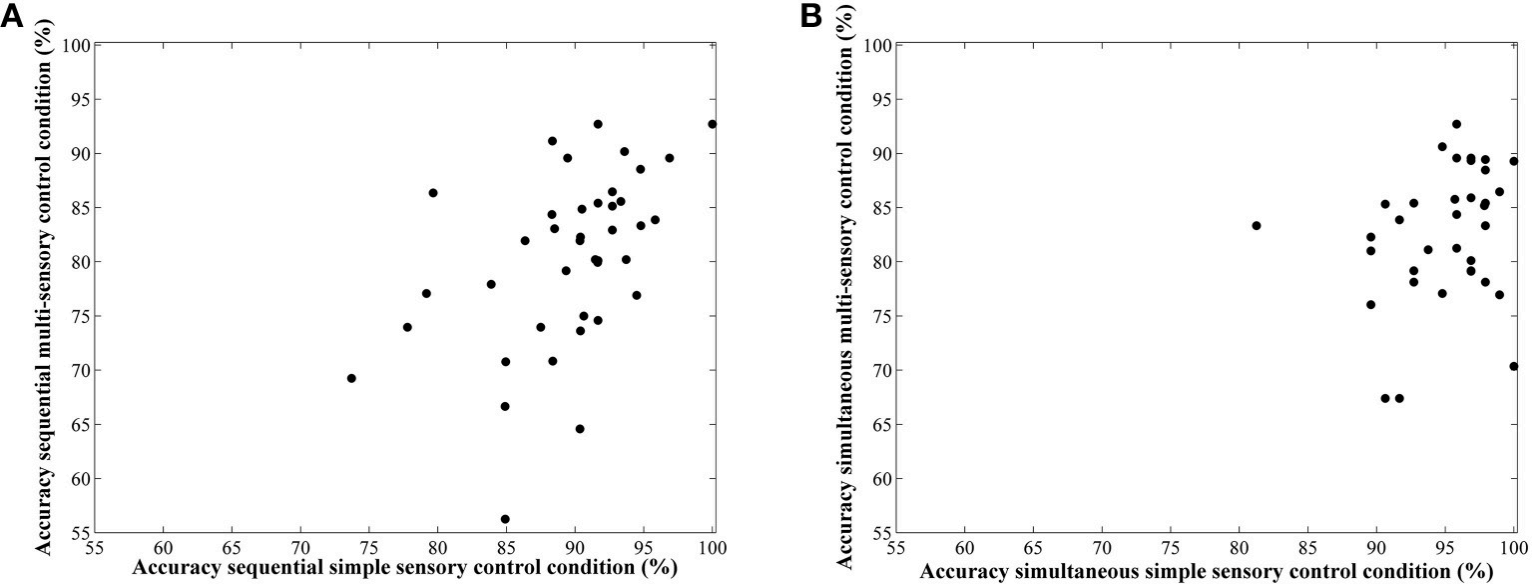
**Scatterplots to illustrate the relationship between accuracy in the simple and multi-sensory control condition in the sequential (A) and simultaneous (B) presentation condition**. The correlation was significantly stronger in the first compared to the latter.

**Figure 7:**
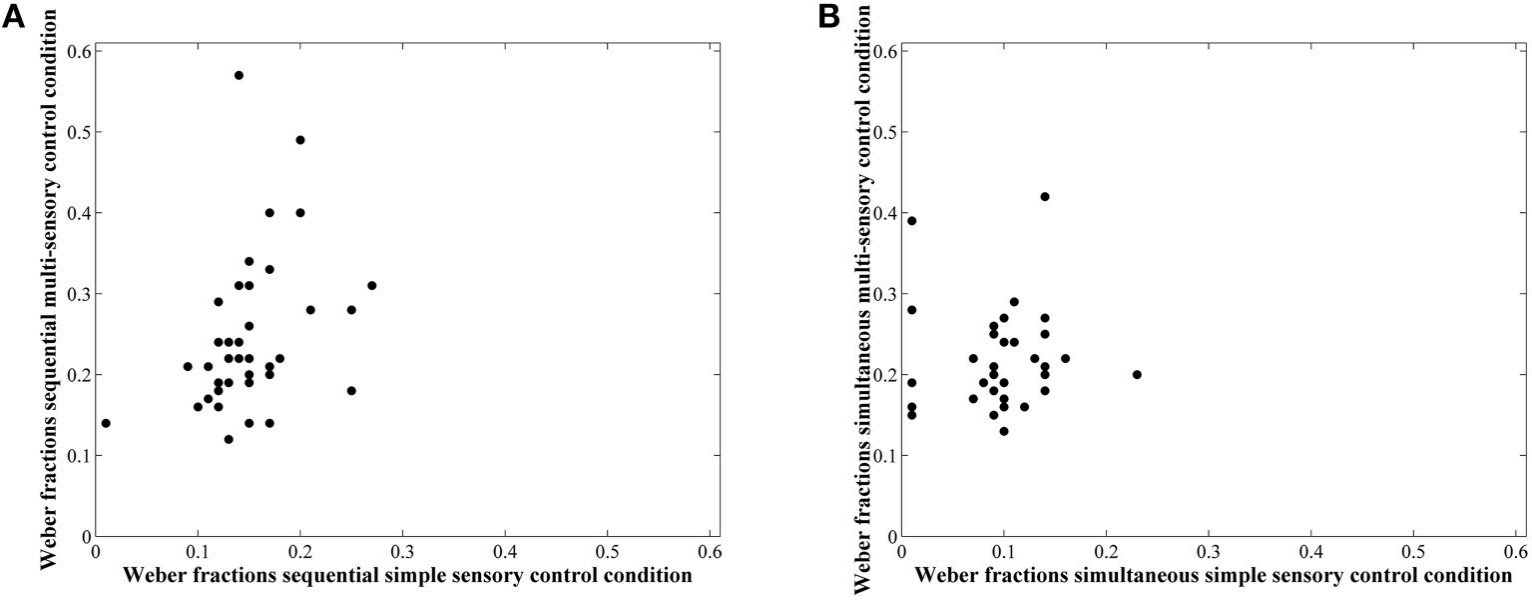
**Scatterplots to illustrate the relationship between Weber fractions of the simple and multi-sensory control condition in the sequential (A) and simultaneous (B) presentation condition**. The correlation was significant in the first, but not in the latter. The difference between these correlations was significant.

Correlations between average accuracies of the four conditions are shown in Table 1. We especially focus on the correlation between the two types of visual cue control within each condition as the aim of our study was to examine the effect of a distinct type of visual cue control in each presentation format. We found there to be a significant correlation between performances on both sequential tasks, *r*_(38)_ = 0.49, *p* = 0.001 (Figure 6A), whereas the correlation between accuracies of the simultaneous conditions did not reach significance, *r*_(38)_ = 0.26, *p* = 0.11 (Figure 6B). However, considering the fact that split-half reliabilities were rather moderate (especially for the simultaneous condition), we adjusted the calculated correlations for reliability (by means of the attenuation formula, Murphy and Davidshofer, 1988). The Pearson-Filon *Z*-test for examining the difference between two non-overlapping correlations in dependent samples indicated that the corrected correlation between both types of visual cue control was marginally significant, *p* = 0.079, indicating a stronger correlation in the sequential, *r*_(38)_ = 0.73, compared to the simultaneous condition, *r*_(38)_ = 0.52 (Figure 6). Correlations between Weber fractions in each of the four conditions are also illustrated in Table 1. Similar to the correlation analyses with accuracies, there was a significant correlation between Weber fractions of the two sequential conditions, *r*_(38)_ = 0.35, *p* = 0.03 (Figure 7A), whereas the correlation between Weber fractions of the simultaneous conditions was not significant, *r*_(38)_ = 0.04, *p* = 0.80 (Figure 7B). We adjusted these calculated correlations for reliability and performed a Pearson-Filon *Z*-test for examining the difference between the two adjusted correlations that were the focus of our research question. This test was highly significant, *p* < 0.001, and indicated that the two correlations between Weber fractions of the two visual cue control conditions within each presentation format were significantly different from one and other: Weber fractions between the two visual cue controls in the sequential conditions were correlated, while Weber fractions between the two visual cue controls in the simultaneous conditions were not related. As we could not control reaction times for general processing speed, analogous correlations for reaction times were not computed.

**Table 1:** **Correlations between the four conditions (with ***p***-values)**.

**Accuracies**	**1**	**2**	**3**	**4**
1. Sequential simple sensory	–			
2. Sequential multi-sensory	0.49 (0.001)	–		
3. Simultaneous simple sensory	0.31 (0.05)	0.42 (0.007)	–	
4. Simultaneous multi-sensory	0.26 (0.11)	0.43 (0.006)	0.25 (0.12)	–
**ADJUSTED ACCURACIES**				
1. Sequential simple sensory	–			
2. Sequential multi-sensory	0.73 (<0.001)	–		
3. Simultaneous simple sensory	0.56 (<0.001)	0.71 (<0.001)	–	
4. Simultaneous multi-sensory	0.47 (0.002)	0.73 (<0.001)	0.52 (<0.001)	–
**WEBER FRACTIONS**				
1. Sequential simple sensory	–			
2. Sequential multi-sensory	0.35 (0.03)	–		
3. Simultaneous simple sensory	0.34 (0.04)	0.15 (0.38)	–	
4. Simultaneous multi-sensory	0.03 (0.84)	0.22 (0.19)	0.04 (0.80)	–
**AJUSTED WEBER FRACTIONS**				
1. Sequential simple sensory	–			
2. Sequential multi-sensory	0.67 (<0.001)	–		
3. Simultaneous simple sensory	0.83 (<0.001)	0.28 (0.08)	–	
4. Simultaneous multi-sensory	0.08 (0.62)	0.44 (0.004)	0.10 (0.54)	–

### *Post-hoc* analyses

As one reviewer insightfully pointed out to us, some participants in the simple sensory control condition performed on an extremely high level (i.e., an accuracy of 100%, see Figure 6). This raised the question whether the visual cue control in this condition was done at a satisfactory level. To evaluate whether visual cues were sufficiently controlled, we conducted Chi square tests with numerosity difference (coded as -1/+1 if N1 smaller/larger than 18, respectively) and all corresponding visual cues (coded as -1/+1 when the difference is smaller/larger than 0 respectively) in both stimulus sets. If, as we expected, the visual cues are properly controlled, the Chi square tests should be non-significant, indicating that the larger numerosity contains the larger visual cues in half of the trials, whereas in the other half, the larger numerosity has smaller visual cues. In the multi-sensory control condition, all Chi square tests were non-significant [all $\chi_{\left(1\right)}^{2}$ < 0.05; *p* > 0.83]. This was also true for in the simple sensory control condition for density, surface and diameter [all $\chi_{\left(1\right)}^{2}$ < 0.69; *p* > 0.40]. However, unexpectedly, Chi square tests between numerosity difference and area extended on the one hand and circumference on the other hand were significant [$\chi_{\left(1\right)}^{2}$ = 51.60; *p* < 0.001 and $\chi_{\left(1\right)}^{2}$ = 96; *p* < 0.001 respectively], indicating a confound between these visual cues and numerosity. This unexpected finding was further explored with separate analyses in both batches of stimuli that result from using the simple sensory control condition. In this program, pairs of numerosities are created in which in half of the trials dot size and area extended (note that the program does not manipulate circumference of the stimuli explicitly, a visual cue also confounded in the present stimulus set. However, the same arguments hold as these two parameters are perfectly correlated in this condition) are at a constant level, while surface co-varies with numerosity. In the other half of the trials, surface is maintained at a constant level, while both area extended and dot size co-vary with numerosity. Overall, this method is assumed to lead to a non-predictive relationship (i.e., no correlation) between the controlled visual cues and numerosity in order to prevent participants from relying on these visual cues to perform the task. This also means that, for instance, for the visual cue area extended, trials can be subdivided in match and non-match trials. In the match trials, area extended of both numerosities in a pair is maintained constant, which should lead to a zero correlation in these trials. In the non-match trials, area extended co-varies with numerosities, thus leading to a significant and positive correlation between area extended and numerosity. To verify the latter correlations in both the match and non-match stimuli, we performed separate *post-hoc* correlational analyses for each batch of stimuli (match vs. non-match) to verify whether the visual cues were predictive of numerosity. The analyses indicated that most visual cues behaved as expected when following the rationale of the script provided by Dehaene et al. (2005) in the respective match (visual cue constant between both numerosities of the pair and no significant correlation) and non-match stimuli (visual cue co-varies with numerosity). In contrast, for the visual cue area extended, this was not the case: The correlation in the non-match or co-varying stimuli was, as expected, very high (*r* = 0.97, *p* < 0.0001). However, in the match stimuli, this correlation was also significant (*r* = 0.73, *p* < 0.0001), indicating a relationship between area extended and numerosity in the stimuli in which area extended was supposed to be at a constant level. These *post-hoc* analyses indicate that in the simple-sensory control condition, participants could have used area extended to respond.

To determine whether this confound also significantly influenced participants' comparison behavior, we fitted separate logistic mixed-effects regression models with either ratio or the absolute difference in area extended as independent predictors of accuracy (Jaeger, 2008) to investigate which of the two predictors yielded the best model fit. We ran these analyses separately rather than including both predictors simultaneously because they were collinear, which can dramatically influence the estimates of the regression weights. Because of convergence issues, both predictors were standardized before fitting the models. As recommended by Barr et al. (2013), a full random effects structure (i.e., random intercept and random slope for the predictors) was used in all models (we did not implement correlations between random effects to reduce model complexity). We estimated four different models, split up by presentation format (simultaneous vs. sequential) and match/non-match trials for the simple sensory control condition.

Table 2 summarizes the results of these analyses. The measures of model fit that are provided are the Aikaike information criterion (AIC), the Bayesian information criterion (BIC) and a conditional *R*^2^ measure. Lower AIC and BIC values indicate better model fit and can be used to directly compare model fit of non-nested models. The conditional *R*^2^ is a recently developed measure to assess the absolute goodness-of-fit of generalized linear mixed-effects models (Nakagawa and Schielzeth, 2013) and takes into account the variance explained by both the fixed and random effects. To compare model fit, however, we rely on which model yields a lower AIC and BIC score. As can be derived from Table 2, in all four cases considered, area as well as ratio were almost equally good predictors of performance. However, in all but one case, the models including ratio as a predictor, yielded a slightly better model fit than the models including area as predictor as indexed by both information criteria (yet note that in all cases considered the conditional *R*^2^ is higher for the model including ratio as a predictor). This pattern of results was somewhat attenuated in the simultaneous condition with the non-match stimuli where area was allowed to co-vary with numerosity. In this case, ratio and area were equally good predictors. In summary, these results indicate that ratio was a slightly better predictor than area for all cases considered, except the one for which it could reasonably be expected that participants extract the co-varying relations between area and numerosity. These results are in line with the observed correlations between conditions and further illustrate an important distinction between the simultaneous and the sequential task and possibly suggest that participants extract the relation between area and numerosity in the case where this relationship is very strong, but only when both stimuli are simultaneously presented and not when they are presented in a sequential fashion.

**Table 2:** *****Post-hoc*** analyses in the simple sensory control condition with separate models including either area extended or ratio and split up by presentation format and match/non-match**.

**Model**	**Estimate**	**Standard error**	***Z*-value**	***p*-value**	**AlC**	**BIC**	**Conditional *R*^2^**
**SIMULTANEOUS MATCH**							
Area model	0.68	0.15	4.48	<0.0001	1264	1286	0.46
Ratio model	0.76	0.11	7.20	<0.0001	1225	1247	0.52
**SIMULTANEOUS NON-MATCH**							
Area model	1.11	0.22	5.04	<0.0001	1013	1035	0.60
Ratio model	0.99	0.17	5.77	<0.0001	1013	1036	0.63
**SEQUENTIAL MATCH**							
Area model	0.28	0.12	2.25	0.02	1428	1450	0.16
Ratio model	0.37	0.09	4.32	<0.0001	1413	1436	0.20
**SEQUENTIAL NON-MATCH**							
Area model	0.57	0.12	4.62	<0.0001	1083	1105	0.22
Ratio model	0.68	0.11	6.34	<0.0001	1068	1089	0.23

## Discussion

It is implicitly assumed in the literature that comparing results across studies with a wide range of differing methodological characteristics is possible (e.g., Halberda and Feigenson, 2008; Piazza et al., 2010). This assumption, however, may not be viable. One methodological aspect that can be differently manipulated refers to the applied type of visual cue control. The results of a previous study (Smets et al., 2015) indicated that an influence of the type of visual cue control was present when participants were required to indicate the larger of two simultaneously presented stimuli. The current study extends upon this previous research by examining whether the influence of distinct types of visual cue control on performance is diminished when presenting stimuli in a numerosity comparison task in a sequential instead of a simultaneous fashion. Since an abundance of different methods to control the visual cues of dot stimuli exist and it is virtually impossible to contrast all of these within-subjects, we chose to include two methods exactly as they are implemented in the literature. These two methods differ markedly in the rigor of visual cue control as they either manipulate only a few visual cues and are assumed to be an appropriate control when participants rely on a single visual cue when comparing numerosities (i.e., simple sensory control method; Dehaene et al., 2005) or manipulate multiple visual cues and are assumed to be an appropriate control when participants rely on an integration of multiple visual cues or switch between visual cues (i.e., multi-sensory control method; Gebuis and Reynvoet, 2011a).

Overall, the results of the present study confirmed our hypothesis: Although the influence of the applied type of visual cue control on accuracy was present both when stimuli were presented simultaneously and sequentially, there was a significantly larger difference between the simple and the multi-sensory control condition in the first compared to the latter presentation format. Furthermore, the same difference between the simple and multi-sensory control condition for reaction times was significant in the simultaneous but not in the sequential condition. In addition, accuracies in the simple and multi-sensory control condition were not significantly correlated when the stimuli were simultaneously presented. When adjusting the correlation between accuracies for reliability, the correlation between the two visual cue control conditions in the simultaneous condition did reach significance, but was markedly less strong compared to the sequential condition. In addition, the large and significant difference in the strength of the relationship between Weber fractions of both visual cue control conditions in the two presentation formats also seems to suggest that distinct visual cue controls have a stronger influence on performance in the simultaneous comparison task. The *post-hoc* analyses that were performed to determine the effect of a confound between area extended and numerosity in the simple sensory control condition, also hint toward a distinction between the sequential and simultaneous task. Although the differences between model fits with ratio and area as predictors were small in all conditions, the results suggested that participants were able to extract the relation between area extended and numerosity (i.e., in the non-match or co-varying stimuli) slightly better in the simultaneous condition and less so in the sequential condition.

Hence, in correspondence with our conclusion from a previous study (Smets et al., 2015), these results suggest that in some instances more than in others, the impact of the applied type of visual cue control is stronger. More concrete, simultaneously presenting participants with two dot arrays permits explicit and refined visual comparisons (Brown and Rebbin, 1970), leading participants to (un)consciously rely on or experience interference from automatically extracted visual cues (e.g., Clearfield and Mix, 2001; Hurewitz et al., 2006; Gebuis and Reynvoet, 2013). This unconscious reliance on or interference from visual cues can potentially increase performance if these cues provide additional and reliable information with respect to numerosity. The two visual cue controls applied in the current study differ markedly in the information visual cues provide with respect to numerosity. More specifically, visual cues in the multi-sensory condition are more ambiguous and provide less information with respect to numerosity, which could either be due to the manipulation of *multiple* visual cues or the heterogeneity of dot sizes within one array. This is explicitly confirmed in the *post-hoc* regression analyses, illustrating that visual cues in the simple sensory control condition may still provide relevant information with respect to numerosity, whereas this was not found to be the case in the multi-sensory control condition. Consequently, a higher performance in the simple sensory control condition (Dehaene et al., 2005) compared to the multi-sensory control condition (Gebuis and Reynvoet, 2011a) is obtained. The observation that numerosity comparison becomes markedly more difficult when stimuli are constructed by means of the multi-sensory control method is in correspondence with the results of Szücs et al. (2013), who also found a decreased performance compared to previous studies which mostly employed simple sensory control.

In the sequential condition however, visual comparison does not occur as simple and efficient as in the simultaneous condition and only the latter visual display can be stored in visuo-spatial short term memory (Frick, 1985), forcing participants to resort to a different strategy. One such strategy could be to focus more strongly on the numerosity aspect of the dot arrays since this aspect is accentuated in the task instructions (i.e., “indicate the larger numerosity”). This process strongly resembles numerosity estimation for which no effect of distinct types of visual cue control was found in a previous study (Smets et al., 2015). As a consequence, visual cues do not interfere as much in the comparison judgment when stimuli are sequentially presented, leading to an effect of distinct visual cue controls.

However, we should be cautious with this interpretation of the data as a consequence of the methodological confound in the simple sensory control condition. In the simple sensory control condition, half of the stimulus pairs have the same dot size and area extended, while surface co-varies with numerosity. In the other half of the trials, surface is maintained constant, while area extended and dot size co-vary with numerosity. Overall, this should lead to a non-predictive relationship between the visual cues and numerosity. However, unexpectedly, *post-hoc* analyses of the visual cues of our stimuli showed that area extended is confounded with number, also in those trials in which it was supposed to be matched. This confound between area extended and number may have allowed participants to focus on area instead of number. If this is the case, it is difficult to compare performance in simple and multi-sensory control conditions as participants may have used different strategies in both conditions. We tried to examine this possibility by regressing either ratio or area extended as predictors of accuracy. Although ratio was a better predictor in most cases, the differences between model fits with ratio and area extended were small and accordingly, our interpretation should be considered as indicative. Additional studies, without the number-area confound in the simple sensory condition are required before firm conclusions can be drawn. This unexpected confound in the simple sensory condition also demonstrates that it is advisable that, in order to make meaningful progress in the debate on the influence of visual cues in numerosity processing, all studies should verify their stimuli for potential confounds after running a stimuli generation program and report the outcome of these verifications.

A potentially relevant note is that the simultaneous and sequential condition not only differed with respect to the presentation format of the stimuli. Another difference between both presentation formats lies in the fact that stimuli in the sequential condition are displayed in the same location, whereas stimuli in our simultaneous condition were positioned side-by-side. This is different from an intermixed presentation format in which the two stimuli are also presented at the same time, but now however also in the same location (e.g., Halberda et al., 2008; Lourenco et al., 2012). Whether our conclusions also hold for intermixed presentation of stimuli should be further investigated in future research. Furthermore, as the results of Inglis and Gilmore (2013) suggested that presentation time impacts performance, shorter or longer presentation durations could potentially also lead to different results.

The effect of distinct types of visual cue control, suggesting an influence of visual cues when participants are required to compare numerosities either in a simultaneous or sequential presentation format, questions the existence of a dedicated ANS functioning *entirely* independently from visual cues (see also Tokita and Ishiguchi, 2013). One possibility is that visual cues are merely integrated to arrive at a numerosity judgment while disregarding numerosity completely (e.g., Gebuis and Reynvoet, 2011b; Tokita and Ishiguchi, 2013). This view implies that participants explicitly use visual cues that co-vary with numerosity. However, the connectionist model of Stoianov and Zorzi (2012) showed that numerosity can in fact be *extracted* independently from visual cues. This implies that observed errors occur at a behavioral level with visual cues for instance still influencing number judgments when participants are unable or less able to inhibit visual cue information adequately (Cappelletti et al., 2014). More specifically, if numerosity and visual cues are processed in parallel, mere interference of visual cues at a behavioral level when performing number judgments could be a consequence (Burr and Ross, 2008; Anobile et al., 2013; Gebuis and Reynvoet, 2014). Considering that numerosity related effects were still obtained in the present study (i.e., ratio effects), the latter possibility seems to be supported. However, further research is necessary to unravel this issue as we cannot distinguish between an explicit use vs. a mere interference of visual cues.

Future research should be encouraged to further disentangle the effects of these and other differences in methodology to come to a full understanding of the ANS and its relationship with several other skills (e.g., mathematics: Halberda et al., 2012; Sasanguie et al., 2012; cardinality knowledge: Rousselle and Noël, 2008; Abreu-Mendoza et al., 2013). In addition, the current results point out that, despite the use of visual cue controls frequently applied in the literature, there still is an interfering influence of visual cues causing the observed difference in performance between distinct visual cue controls. However, since it is physically impossible to control all visual cues, a different approach may be necessary. For instance, by introducing a large variability in visual cues and only small differences in these visual cues between numerosities, there may still be a strictly physically speaking relationship between numerosity and certain visual cues, but the differences may not distinguishable for participants on an individual level. Consequently, these visual cue are still unusable predictors to indicate numerosity. Psychophysical studies investigating this issue are however necessary to assess what differences between visual cues are not noticeable for participants.

To conclude, sequentially presenting the stimuli in a numerosity comparison task did not entirely exclude the influence of the applied type of visual cue control: Participants in both the sequential and simultaneous presentation conditions performed significantly better when only a few visual cues were controlled in a relatively simple manner (Dehaene et al., 2005) compared to a more stringent method of controlling multiple visual cues (Gebuis and Reynvoet, 2011a). However, using a simultaneous presentation in numerosity comparison induced a larger influence of the type of visual cue control and a significant reliance on area extended in the simple sensory control condition compared to the sequential design. This indicates that certain methodological aspects of the latter design do in fact diminish the influence of distinct types of visual cue control. In the simultaneous condition, visual cue comparison happens with great ease and can be performed rather explicitly as both stimuli are presented together on the screen. In the sequential presentation condition however, direct visual cue comparison is not possible and strategies similar to those in numerosity estimation may be used as a means to complete the task, thus reducing the influence of distinct visual cue controls. More general, the results of the present study showed that methodological differences in the type of visual cue control that is applied can lead to instable and potentially unrelated performances, both when presenting stimuli simultaneously and sequentially (see also Smets et al., 2015) Given these results, caution is necessary, especially considering the frequent use of the comparison task and the wide range of different types of visual cue control of which the currently contrasted methods are merely two.

### Conflict of interest statement

The authors declare that the research was conducted in the absence of any commercial or financial relationships that could be construed as a potential conflict of interest.
